# Cognitive reserve, depressive symptoms, obesity, and change in employment status predict mental processing speed and executive function after COVID-19

**DOI:** 10.1007/s00406-023-01748-x

**Published:** 2024-01-29

**Authors:** Mar Ariza, Javier Béjar, Cristian Barrué, Neus Cano, Bàrbara Segura, NAUTILUS Project Collaborative Group, Jose A Bernia, Vanesa Arauzo, Marta Balague-Marmaña, Cristian Pérez-Pellejero, Silvia Cañizares, Jose Antonio Lopez Muñoz, Jesús Caballero, Anna Carnes-Vendrell, Gerard Piñol-Ripoll, Ester Gonzalez-Aguado, Mar Riera-Pagespetit, Eva Forcadell-Ferreres, Silvia Reverte-Vilarroya, Susanna Forné, Jordina Muñoz-Padros, Anna Bartes-Plan, Jose A. Muñoz-Moreno, Anna Prats-Paris, Inmaculada Rico Pons, Judit Martínez Molina, Laura Casas-Henanz, Judith Castejon, Maria José Ciudad Mas, Anna Ferré Jodrà, Manuela Lozano, Tamar Garzon, Marta Cullell, Sonia Vega, Sílvia Alsina, Maria J. Maldonado-Belmonte, Susana Vazquez-Rivera, Eloy García-Cabello, Yaiza Molina, Sandra Navarro, Eva Baillès, Claudio Ulises Cortés, Carme Junqué, Maite Garolera

**Affiliations:** 1 Grup de Recerca en Cervell, Cognició i Conducta, Consorci Sanitari de Terrassa (CST), Terrassa, Spain; 2 Unitat de Psicologia Mèdica, Departament de Medicina, Universitat de Barcelona (UB), Barcelona, Spain; 3 Departament de Ciències de la Computació, Universitat Politècnica de Catalunya-BarcelonaTech, Barcelona, Spain; 4 Departament de Ciències Bàsiques, Universitat Internacional de Catalunya (UIC), Sant Cugat del Vallès, Spain; 5 Institut d’Investigacions Biomèdiques August Pi i Sunyer (IDIBAPS), Barcelona, Spain; 6 Institut de Neurociències, Universitat de Barcelona (UB), Barcelona, Spain; 7 Neuropsychology Unit, Consorci Sanitari de Terrassa (CST), Terrassa, Spain

**Keywords:** Post-COVID-19 condition, Mental speed processing, Executive function, Machine learning, Clustering, Logistic regression

## Abstract

The risk factors for post-COVID-19 cognitive impairment have been poorly described. This study aimed to identify the sociodemographic, clinical, and lifestyle characteristics that characterize a group of post-COVID-19 condition (PCC) participants with neuropsychological impairment. The study sample included 426 participants with PCC who underwent a neurobehavioral evaluation. We selected seven mental speed processing and executive function variables to obtain a data-driven partition. Clustering algorithms were applied, including K-means, bisecting K-means, and Gaussian mixture models. Different machine learning algorithms were then used to obtain a classifier able to separate the two clusters according to the demographic, clinical, emotional, and lifestyle variables, including logistic regression with least absolute shrinkage and selection operator (LASSO) (L1) and Ridge (L2) regularization, support vector machines (linear/quadratic/radial basis function kernels), and decision tree ensembles (random forest/gradient boosting trees). All clustering quality measures were in agreement in detecting only two clusters in the data based solely on cognitive performance. A model with four variables (cognitive reserve, depressive symptoms, obesity, and change in work situation) obtained with logistic regression with LASSO regularization was able to classify between good and poor cognitive performers with an accuracy and a weighted averaged precision of 72%, a recall of 73%, and an area under the curve of 0.72. PCC individuals with a lower cognitive reserve, more depressive symptoms, obesity, and a change in employment status were at greater risk for poor performance on tasks requiring mental processing speed and executive function. *Study registration***:** www.ClinicalTrials.gov, identifier NCT05307575.

**Supplementary Information:**

The online version contains supplementary material available at 10.1007/s00406-023-01748-x.

## Introduction

Post-COVID-19 condition (PCC) develops in people previously infected with the SARS-CoV-2 virus, approximately 3 months after infection. These individuals exhibit symptoms that persist for no less than 2 months and cannot be accounted for by another diagnosis [1]. Typical symptoms include, though are not exhaustively limited to, fatigue, difficulty breathing, and cognitive impairment, which tend to adversely affect daily life activities.

The prevalence of PCC varies according to the study cohort, the methodology, the SARS-CoV-2 variant, and the vaccination status [2]. Nevertheless, according to a prevalence study that accounted for confounding variables, approximately one in eight individuals infected with COVID-19 could develop PCC [3]. Several mechanisms have been suggested to explain the pathophysiology of PCC, including organ damage occurring during the acute phase of infection, a chronic inflammation state, persistent SARS-CoV-2 viral antigens, and reactivation of latent herpesviruses [4, 5].

Risk factors for PCC are heterogeneous and remain poorly characterized. In a recent meta-analysis, Tsampasian et al. [6] reported that female gender, advanced age, and obesity were associated with an increased risk of developing PCC. Other comorbidities, such as anxiety and/or depression, asthma, chronic obstructive pulmonary disease, diabetes, ischemic heart disease, and immunosuppression, were also substantially associated with an increased risk of PCC. In addition, patients who required hospitalization or those in intensive care during the acute phase of COVID-19 infection had a risk of developing PCC that was more than double that of those who did not [6]. In contrast, a healthy lifestyle before infection is associated with a substantially lower risk of PCC, according to a prospective cohort study involving 32,249 women [7]. The risk of presenting persistent neurological symptoms (fatigue, headache, altered smell/taste, and cognitive complaints) was found to be increased in women, those of Hispanic ethnicity, low socioeconomic status, and hospitalization for COVID-19 [8].

The concept of post-COVID cognitive dysfunction has emerged as a potential form of cognitive impairment that occurs at least 3 months after acute infection with COVID-19. This condition is characterized by a distinct cognitive profile, primarily marked by deficits in attention and processing speed. In some cases, these deficits may be accompanied by impairments in executive function and episodic memory [9–11]. Mental processing speed and executive function are highly important for mental and physical health and in achieving success in academic and personal goals [12]. Mental processing speed refers to an individual’s capacity to process information efficiently and rapidly, and it is highly correlated with higher order cognitive functions [13]. Executive function regulates behavior and other cognitive processes, including attention and memory [14]. Alterations in executive function and speed processing can have significant adverse effects on daily functioning [15, 16].

Several factors are associated with post-COVID cognitive dysfunction. The severity of COVID-19 has been thoroughly investigated, yielding mixed results [17]. Further risk factors for cognitive impairment include age [9, 18, 19], level of education [9, 20–22], sex [19, 23], and the presence of comorbidities, particularly obesity [19]. On the other hand, cognitive reserve (CR) is the most significant protective factor for post-COVID cognitive impairment [24–27]. However, the existing body of research on CR has primarily focused on post-COVID patients who have undergone treatment in the intensive care unit (ICU) [24–27] or during the subacute phase [24, 26].

Machine learning techniques utilized across diverse fields of health can be categorized into unsupervised and supervised learning. Unsupervised learning refers to a learning algorithm that does not rely on labeled data and aims to partition data points into homogeneous groups to find a dataset’s natural structure and underlying patterns using a measure of similarity [28]. Several studies have categorized patients into groups, and distinct forms of cognitive dysfunction have been identified solely based on the patient’s data through unsupervised clustering techniques [29–32]. Supervised learning is used to train classification and prediction models using examples or outputs given a set of labels or a continuous response [33]. The utilization of supervised machine learning algorithms for disease prediction has become increasingly popular [34]. Cognitive and neuroimaging data have proven valuable in predicting several neurological and psychiatric conditions [35–38].

Unsupervised machine learning clustering techniques have been tested to identify post-COVID-19 cognitive phenotypes. Although data from five different cognitive domains were used, only two clusters emerged that differed in cognitive impairment according to the study of Matias-Guiu et al. [9]. In the present study, we focus on tests that measure mental processing speed and executive function, which we had found to be altered in our previous research [39]. We propose that an unsupervised partition of participants based on cognitive performance could be obtained using clustering algorithms. Then using a new set of variables and a supervised learning algorithm, this partition can be characterized by analyzing the characteristics of different groups and the relevance of the selected variables in the group separation.

The aim of this study was to identify the sociodemographic, clinical, and lifestyle characteristics that characterize a group of PCC participants with neuropsychological impairment.

## Materials and methods

### Participants

We conducted an observational, exploratory multicenter study of patients enrolled from neuropsychology and COVID-19 units across 19 hospitals in Catalonia, Madrid, Canarias (Spain), and Andorra, coordinated by the Consorci Sanitari de Terrassa (Barcelona, Spain) (ClinicalTrials.gov ID NCT05307575). We included preliminary data on 426 participants with PCC recruited from June 2021 through to July 2023. The mean age of the participants was 50.29 years (SD = 9.5), the mean level of formal education was 13.94 years (SD = 3.33), and 285 of them (70%) were female. Participants with PCC were classified into three groups according to the WHO clinical progression scale [40]: severe-intensive care unit (ICU) (6 to 9 points), hospitalized (4 and 5 points), and mild (2 and 3). According to the severity of the disease, 182 (42.7%) participants were hospitalized, of which 96 (22.5% of total sample) were admitted to the intensive care unit (ICU). The remaining 244 (57.3%) individuals with PCC were outpatients and had a mild illness at home. The mean time since onset to assessment was 362 (SD = 229) days. The age, sex, level of education, and severity of COVID-19 did not differ substantially among participants recruited at each center; however, the duration between the onset of illness and the time of assessment exhibited greater heterogeneity (please refer to Supplementary Table 1).

The inclusion criteria for the PCC group were as follows: (a) confirmed diagnosis of COVID-19 according to World Health Organization criteria, with signs and symptoms of the disease during the acute phase; (b) a post-infection period of at least 12 weeks; and (c) aged between 18 and 65 years. The exclusion criteria were as follows: (a) established diagnosis of psychiatric, neurological, or neurodevelopmental disorders, or systemic pathologies known to cause cognitive deficits, prior to COVID-19 infection; and (b) motor or sensory alterations that could impede neuropsychological examinations. In addition, patients who scored < 14 points on the Montreal Cognitive Assessment (MoCA) [41] as a general cognitive screening tool and/or < 85 points on the Word Accentuation Test (TAP) [42] as an estimate of premorbid IQ were excluded. All participants were native Spanish speakers.

Participant anonymity and confidentiality were guaranteed. The study was conducted with the approval of the Drug Research Ethics Committee (CEIm) of Consorci Sanitari de Terrassa (CEIm code: 02–20-107–070) and the Ethics Committee of the University of Barcelona (IRB00003099). The investigation followed the latest version of the Declaration of Helsinki.

### Procedure

Before participation, written informed consent was obtained from all participants. Participation in the study was voluntary, and all participants followed a standardized protocol to achieve their participation requirements.

In the first session, we collected data on sociodemographic characteristics: age, sex, education, income, and change in employment status. The variable change in employment status was collected dichotomously through responses to the following question: Has your current employment situation changed compared to before the illness? We also asked about the employment situation at the time of the evaluation (disability, medical leave, change of job, etc.). In addition, we gathered data on the professions they held before the onset of the illness and classified them into occupational categories following the International Standard for Occupational Classification [43]. We also obtained clinical information: previous comorbidities, COVID-19 symptoms (how they experienced COVID-19), and post-COVID-19 symptoms. For each of 29 typical acute COVID-19 symptoms, the current presence/absence was detailed. Also, we asked participants to report any other symptoms they had been experiencing and had not been covered in the interview. A post-symptom score was calculated by adding the number of persistent symptoms at the time of evaluation and dividing by the total number of possible symptoms. Emotional symptoms were excluded to avoid overlap with the variables collected in the mental health questionnaires. Participants were then scheduled for a second visit in which the neuropsychological examination was performed, as described in Ariza et al. [39]. For this study, we used the following neuropsychological tests: parts A and B of the Trail Making Test (TMT) [44] (visual scanning, motor speed and attention, and mental flexibility); the Controlled Oral Word Association Test (COWAT) [45] (verbal fluency); Digit Symbol Coding Test (WAIS-III) [46] (visual scanning, tracking, and motor speed), and the Stroop Color and Word Test (SCWT) [47] that measured processing speed (SCWT word and color) and cognitive inhibitory control (SCWT word-color). These instruments are recommended for evaluating post-COVID cognitive impairment [48], and prior research has demonstrated their sensitivity to changes in executive function and mental processing speed [17, 49, 50]. All evaluations were performed by trained neuropsychologists.

Finally, participants were provided with all the questionnaires to complete online or on paper to assess different variables. Anxiety symptoms were assessed with the Generalized Anxiety Disorder (GAD-7) [51], depressive symptoms with the Patient Health Questionnaire (PHQ-9) [52], posttraumatic stress symptoms with the Posttraumatic Stress Disorder Checklist for DSM-5 (PCL-5) [53], and fatigue with the Chalder Fatigue Questionnaire (CFQ) [54]. The International Physical Activity Questionnaire (IPAQ) [55] was used to assess physical activity levels, the UCLA Loneliness Scale for loneliness [56], and the Cognitive Reserve Questionnaire (CRC) [57] for CR.

### Statistical and machine learning analyses

Categorical variables were presented as numbers with percentages and continuous variables were presented as means and standard deviations. Differences between clusters were determined using the independent Student’s *t* test and Pearson's Chi-squared test, as appropriate. Odds ratios (ORs) for logistic regression were reported. Analyses were conducted using Python language with the following libraries: Pandas, Scikit-learn, Statsmodels, and SciPy.

**Fig. 1:**
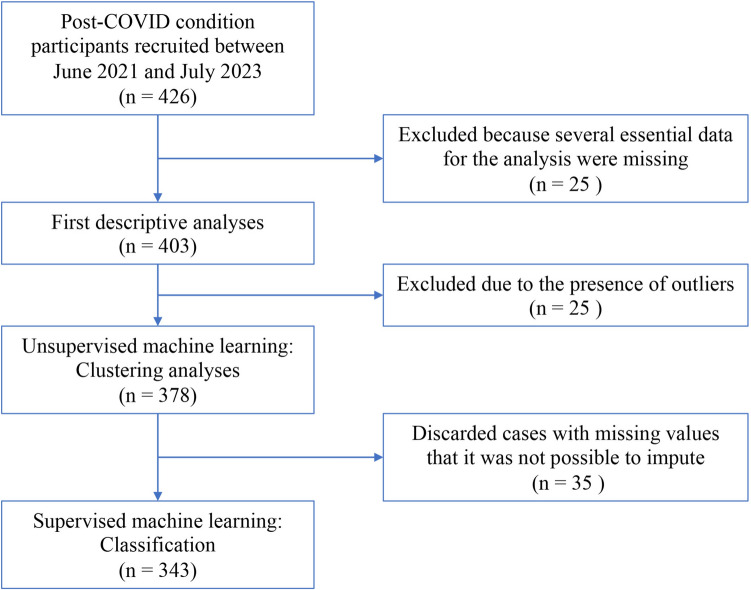
Flowchart for data analysis

The flow chart for data analysis is shown in Fig. 1. The first analysis was performed on a subset of the collected data. We focused on tests that measured processing speed and executive function, cognitive domains in which we had previously observed differences between individuals with PCC and controls without COVID-19 [17, 39]. Seven neuropsychological variables were selected to obtain a partition of the data according to their joint distribution.

Before data analysis, standard preprocessing of the data was applied. The missing values preprocess was applied and those participants with missing values were removed from the analysis. Outlier participants were detected using the local outlier factor (LOF) algorithm [58]. Data were standardized before the analysis and partitioning was performed using partitional clustering. Different clustering algorithms were applied to the data, including K-means, bisecting K-means, and Gaussian mixture models. The number of clusters was assessed using three cluster validity measures (Calinski–Harabasz, Davis–Bouldin, and Silhouette index). Principal component analysis (PCA) was also used to visually assess the validity of the clusters. Logistic regression was computed using the cluster partition as labels to assess the separability of the clusters. Data were divided into training and test sets (70% and 30%), and logistic regression was computed using least absolute shrinkage and selection operator (LASSO) regularization.

For the second analysis, the sociodemographical, clinical, and lifestyle variables were selected. Similar preprocessing of the data was applied using missing values imputation when possible. Data were split into the training and test sets (80% and 20%). Different machine learning algorithms were used to obtain a classifier able to separate the two clusters according to the selected variables, including logistic regression with LASSO (L1) and Ridge (L2) regularization, support vector machines (linear/quadratic/ radial basis function (RBF) kernels), and decision tree ensembles (random forest/gradient boosting trees). Hyper-parameters of the models were adjusted using Bayesian optimization search and tenfold cross-validation. The final model was selected according to accuracy, recall, precision, and the area under receiver operating characteristic curve (AUC). Recursive backward elimination was applied, which reduced the variables of the best model according to the order of the significance using the z-test, and keeping variables with *p* < 0.05. Interpretation of the final model was performed by computing the Shapley values using the SHAP algorithm. This is a well-established interpretation procedure for machine learning methods. Shapley values [59] is a method for assessing variables’ relevance in a model based on the coalitional game theory. It determines the fair payout that corresponds to the participant according to their contribution to the result. In the case of a machine learning model, the value for each variable corresponds to the average contribution of the variable to the classification of the examples in the dataset by the model.

## Results

### Sociodemographic and clinical characteristics

Four-hundred five individuals were employed before acquiring the illness. “Accounting, administrative, and other office employees” (22.7%) comprised the largest occupational category, while “technicians and scientific and intellectual health and teaching professionals” (17.9%) ranked second. “Elementary occupations: unskilled workers in services” and “directors and managers” followed with comparable percentages (9.8% and 9.1%, respectively). In total, 188 PCC individuals (44.7%) underwent employment status changes due to the disease. No significant variations were observed in the proportion based on occupational classification. However, it should be noted that the category “drivers and mobile-plant operators” showed the highest rate of job change (82%), and “qualified workers in agriculture, forestry, and fishing industry” showed the lowest rate of (20%), although both comprised a small number of workers (see Supplemental Table 2 and 3).

Table 1 shows symptoms reported by participants with PCC at the time of assessment. The most frequently reported general symptoms following COVID-19 were fatigue, headache, and pain. On the other hand, the neuropsychiatric symptoms that were reported most frequently were cognitive complaints and manifestations of depression and anxiety.

**Table 1:** Post-COVID-19 participants reported symptoms at the time of assessment

Symptom	*N* (%)
Fatigue	260 (61%)
Headache	191 (44.8%)
﻿Pain	174 (40.8%)
Cognitive complains (subjective)	158 (37.1%)
﻿Dyspnea on exertion	146 (34.3%)
Limb weakness	131 (30.8%)
Depressive symptoms	122 (28.6%)
Anxiety	112 (26.3%)
Cough	111 (26.1%)
﻿Dizziness	109 (25.6%)
﻿Altered smell	106 (24.9%)
Paresthesia	103 (24.2%)
Chest pain	93 (21.8%)
﻿Altered taste	75 (17.6%)
Shaking chills	69 (16.2%)
Posttraumatic stress	51 (12%)
Difficulty sleeping	51 (12%)
Nasal congestion	48 (11.3%)
Sore throat	47 (11%)
Loss of appetite	39 (9.2%)
Nausea	38 (8.9%)
Conjunctival congestion	37 (8.7%)
Diarrhea	37 (8.7%)
Loss of hair	35 (8.2%)
Dermatologic issues	34 (8%)
Tachycardia	32 (7.5%)
Obsessive–compulsive symptoms	16 (3.8%)
Menstrual cycle alteration^a^	10 (10.1%)
Psychotic symptoms	8 (1.9%)

^a^Women up to 47 years (*n* = 109)

### Clustering analyses

For the first analysis, after applying preprocessing to the data, the number of participants included in the dataset to be analyzed was 378. Exploration of the range of the possible number of clusters (2–10) with all the selected clustering algorithms resulted in an agreement on the detection of only two clusters in the data. The obtained partitions were similar; the one obtained with K-means was chosen as it was more natural for computing centroids and obtaining an interpretation of the clusters.

**Fig. 2:**
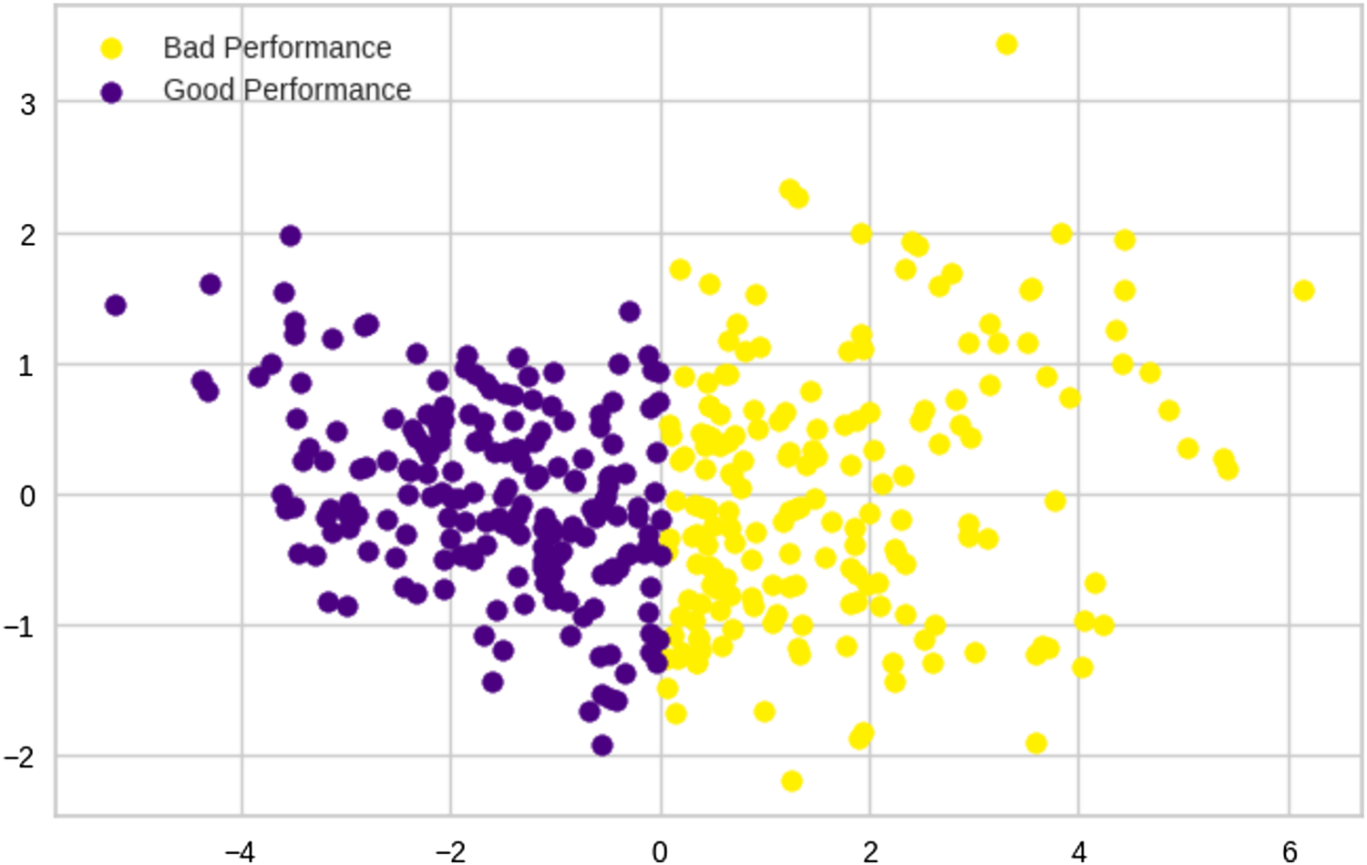
Principal components of the clusters obtained by K-means on the neurophysiological variables

**Fig. 3:**
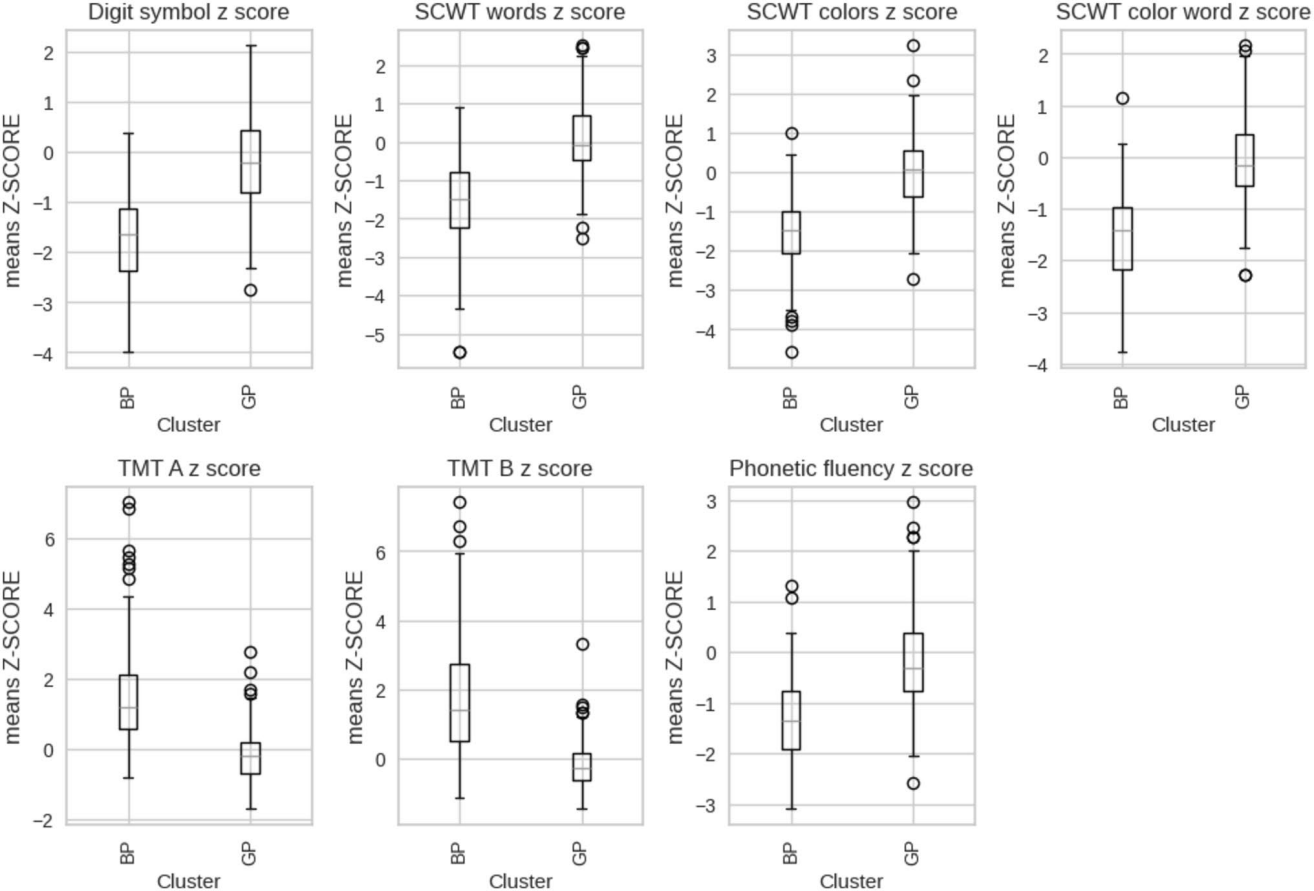
Boxplots grouped by group for each neuropsychological variable. The values ​​of the cognitive tests are standardized with respect to a healthy control group. *BP* bad performance, *GP* good performance, *SCWT* Stroop Color and Word Test, *TMT* = Trail Making Test

The results were also assessed visually by applying PCA to the data; the first two principal components (Fig. 2) corresponded to 69.7% of the total variance of the data. In the figure, two overlapping data clouds can be observed with different distributions. According to the variables’ centroids, we labeled the clusters as good performance (GP) and bad performance (BP). Table 2 shows the means and standard deviations of raw scores of neuropsychological variables corresponding to each cluster. Figure 3 shows a cluster boxplot for each standardized neuropsychological variable.

**Table 2:** Neuropsychological variables for the post-COVID-19 condition (PCC) clusters

	BP	GP
	*n* = 184 Mean (SD)	*n* = 194 Mean (SD)
Digit symbol	52.163 (13.157)	75.278 (13.984)
SCWT words	80.989 (17.068)	104.820 (13.156)
SCWT colors	54.995 (10.279)	71.732 (10.341)
SCWT color word	30.728 (7.956)	45.222 (8.704)
TMT A	44.609 (14.226)	27.954 (7.698)
TMT B	109.815 (41.607)	60.613 (15.837)
Phonetic fluency	33.750 (9.628)	46.588 (11.067)

All variable values are row scores

*BP* bad performance, *GP* good performance, *SCWT* Stroop Color and Word Test, *TMT* = Trail Making Test

**Fig. 4:**
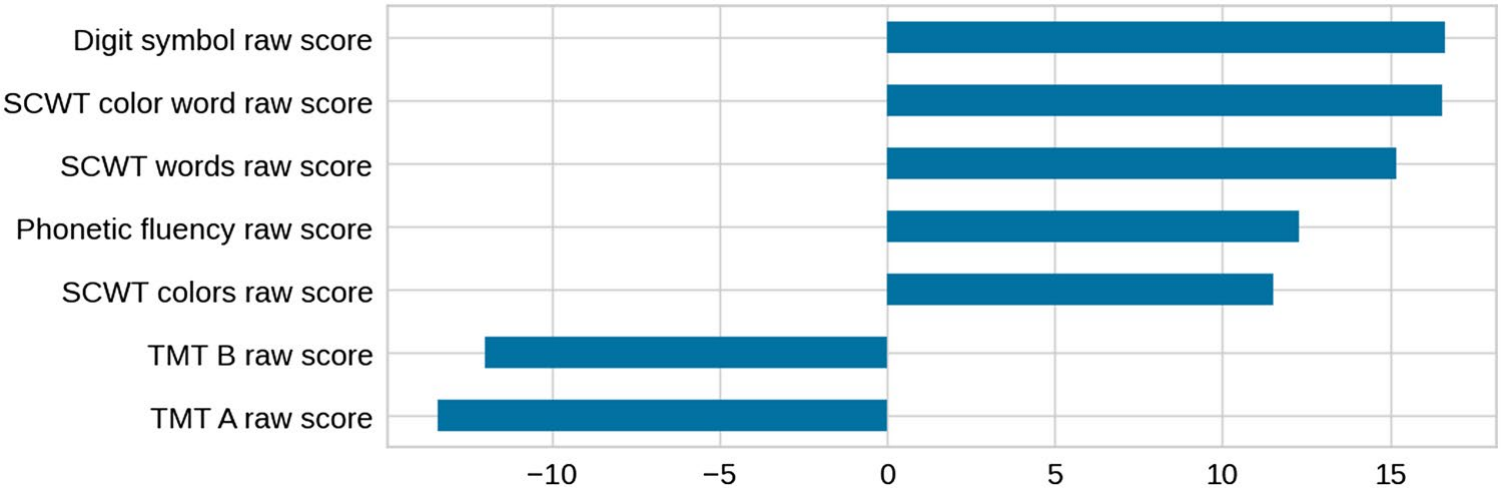
Weights assigned to each one of the attributes in the logistic regression. All variables are used by the logistic regression and have a similar range of weights, with a sign according to the influence on the corresponding classes. *SCWT* Stroop Color and Word Test, *TMT* = Trail Making Test

As the variables did not show non-linear dependencies and the groups were clearly visible using PCA, we applied logistic regression analysis. The accuracy was 99%, with a weighted average 99% recall, 99% precision, and an AUC of 1.0. The weights assigned to each one of the attributes are shown in Fig. 4. The obtained logistic regression was finally used to label the original dataset and to define the partition for further study.

### Classification analyses

For the second analysis, a new dataset with sociodemographical, clinical, and lifestyle variables was defined (Table 2). The original dataset was reduced to 343 participants. The means and standard deviations of the two clusters using the selected variables are shown in Table 3.

**Table 3:** Sociodemographic, clinical, and lifestyle characteristics for the post-COVID-19 condition (PCC) clusters

	BP	GP		
	*n* = 171 Mean (SD)	*n* = 172 Mean (SD)	*t*	*p*
Age (years)	51.32 (8.26)	46.55 (9.52)	4.96	< 0.001
CRC	13.78 (3.34)	15.57 (2.93)	– 6.87	< 0.001
CFQ total score	7.62 (3.64)	5.90 (4.10)	4.11	< 0.001
Days since positive test	336.67 (187.69)	324.27 (174.08)	0.63	0.526
^a^Post-COVID symptoms sum	6.00 (4.48)	3.92 (3.66)	4.69	< 0.001
IPAQ METs total	2335.58 (3556.63)	2803.04 (4020.39)	– 1.14	0.255
GAD-7 score	7.78 (5.57)	5.45 (4.92)	4.10	< 0.001
PCL-5 score	23.53 (17.8)	15.47 (14.69)	4.57	< 0.001
UCLA score	29.25 (8.16)	32.29 (7.02)	– 3.7	< 0.001
PHQ-9 score	11.01 (6.39)	7.49 (6.13)	5.2	< 0.001
	*N* (%)	*N* (%)	*ϰ*^2^	*p*
Sex (female)	107 (62.57)	116 (67.44)	0.69	0.405
Severity			10.49	0.005
ICU	41 (23.97)	30 (17.44)		
Hospitalized	48 (28.07)	30 (17.44)		
Mild	82 (47.95)	112 (65.11)		
Comorbidities				
Respiratory disease	23 (13.45)	22 (12.79)	0.0	0.983
High blood pressure	27 (15.79)	21 (12.20)	0.64	0.423
Dyslipidemia	28 (16.37)	21 (12.20)	0.9	0.343
Obesity	67 (39.18)	29 (16.86)	20.1	< 0.001
Gross salary per year (€)			8.75	0.067
Up to 14K	52 (30.40)	23 (13.37)		
Up to 24K	49 (28.65)	51 (29.65)		
Up to 34 K	45 (26.31)	50 (29.06)		
Up to 39K	12 (7.17)	20 (11.62)		
> 39K	13 (7.60)	28 (16.27)		
Change in employment status	85 (49.7)	45 (26.16)	19.21	< 0.001

*ICU*  intensive care unit, *CRC*  Cognitive Reserve Questionnaire, *CFQ*  Chalder Fatigue Questionnaire, *IPAQ*  International Physical Activity Questionnaire, *GAD-7*  General Anxiety Disorder-7, *PCL-5*  Posttraumatic Stress Disorder Checklist for DSM-5, *UCLA*  UCLA Loneliness Scale, Patient Health Questionnaire

^a^Calculated by adding the number of persistent symptoms at the time of evaluation and dividing by the total number of possible symptoms. Emotional symptoms were excluded to avoid overlap with the variables collected in the mental health questionnaires

All the different machine learning models that were trained had similar results; hence, we choose logistic regression with LASSO regularization for simplicity and interpretability, since it obtains a sparse solution that discards attributes that are not predictive. The accuracy of the model on the test dataset was 74% with a weighted average precision of 74%, recall of 74%, and an AUC of 0.74 (CI 95%: 0.63–0.84). The regularization discarded eight of the chosen variables (coefficients with zero value) and only four remaining variables had coefficients that were significative (*p* < 0.05 according to a *z*-test for the coefficients). The discarded variables were eliminated, and a recursive feature elimination procedure was applied, eliminating variables with less significant coefficients until all the remaining variables were significant (*p* < 0.05). The final model had four of the original variables with an accuracy of 72%, a weighted average precision of 72%, a recall of 73% and an AUC of 0.72 (CI 95%: 0.62–0.83) on the test dataset. This is not a perfect model, but it can be considered reasonably good, explaining a large amount of the behavior of the groups. Table 4 presents the ORs of the variables selected in the logistic regression model.

**Table 4:** Odds ratios (ORs), 95% confidence intervals (95% CIs), and *p* values from the selected variables by the logistic regression model

	OR	95% IC	*p*
CRC score	1.774	1.345–2.341	< 0.001
Change in employment status	0.734	0.560–0.964	0.026
Obesity	0.723	0.555–0.944	0.017
PHQ-9 score	0.712	0.540–0.935	0.016

*CRC* Cognitive Reserve Questionnaire, *PHQ-9* Patient Health Questionnaire

**Fig. 5:**
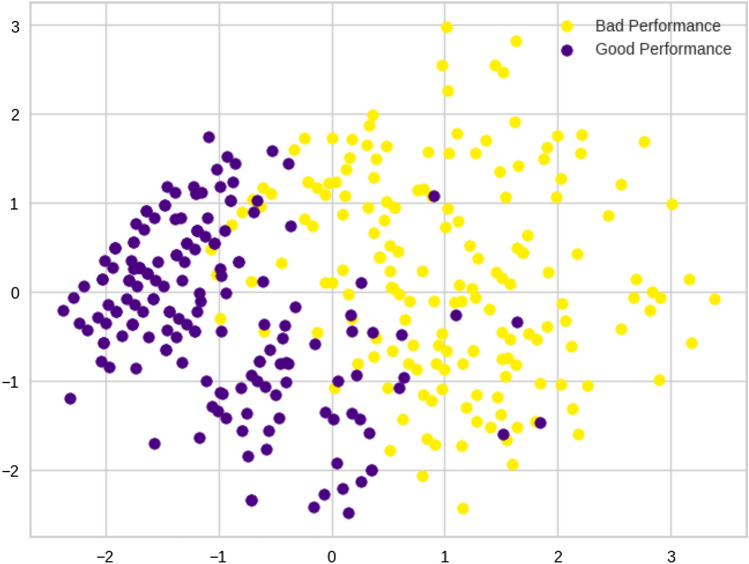
Principal components of the clusters obtained by *K*-means on the sociodemographic, clinical, and lifestyle characteristics selected by the logistic regression model

**Fig. 6:**
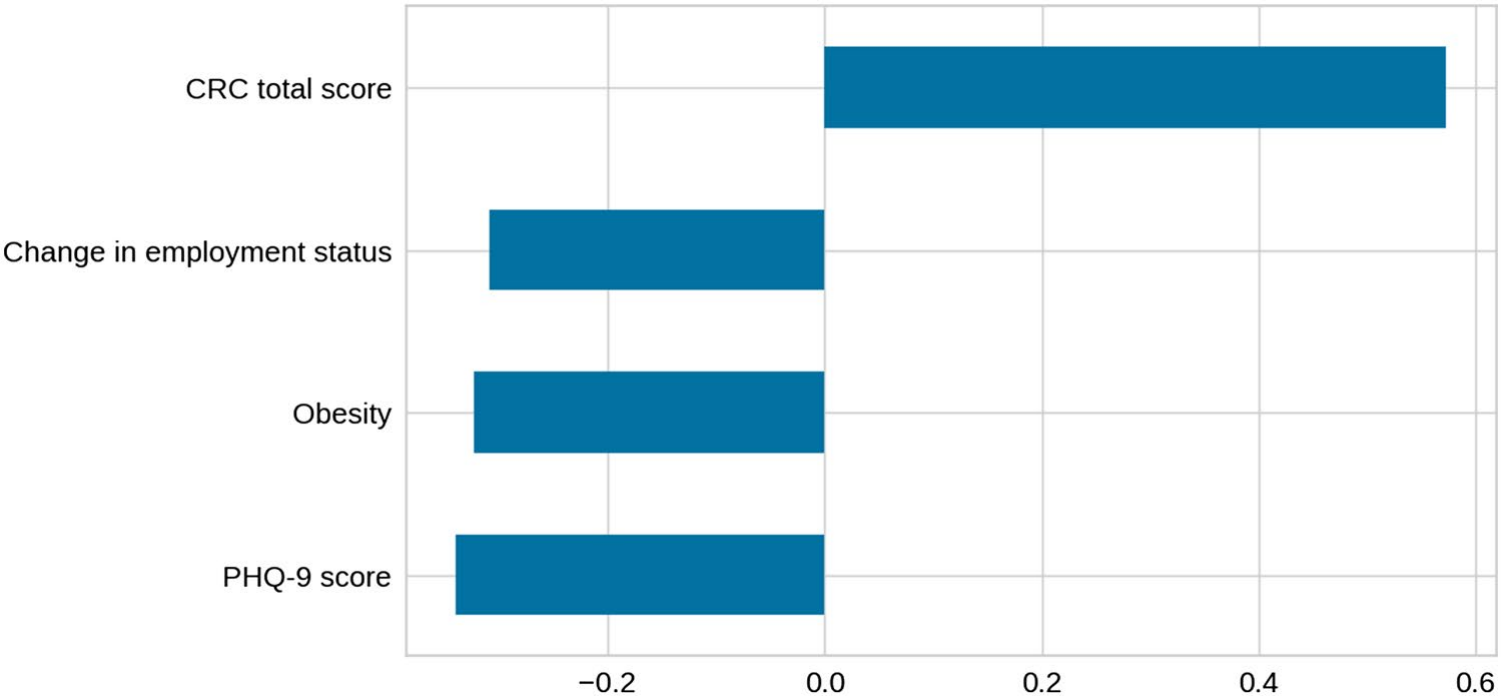
Weights assigned to the selected variables by the logistic regression model. The sign of the weights represents the impact of the variable on the prediction of each of the classes (positive leans to GP class, negative leans to BP class). *GP* good performance cluster, *BP* bad performance cluster, *CRC* Cognitive Reserve Questionnaire, *PHQ-9* Patient Health Questionnaire-9

We applied PCA to the dataset reduced to the four selected variables. The representation of the first two components, which account for 58.6% of the variance, shows a clear separation among the examples from the two classes (Fig. 5). A first interpretation of the logistic regression model can be obtained from the model weights in Fig. 6.

**Fig. 7:**
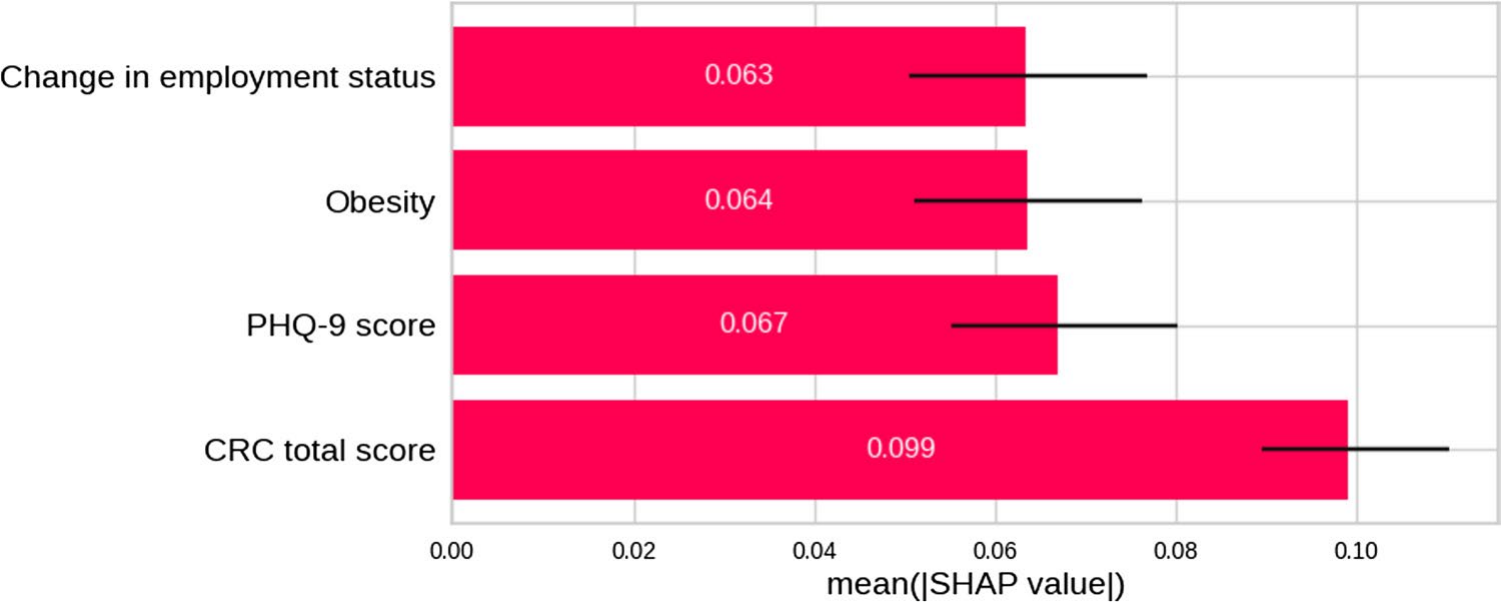
Mean Shapley values computed on the test set for each variable in the model. This represents the mean effect in the absolute value of each variable, that is, the expected change from the mean prediction (probability 0.5) of each variable. *CRC* Cognitive Reserve Questionnaire, *PHQ-9* Patient Health Questionnaire-9. The Shapley values for the model compute the mean of the influence of each variable for changing the class from the mean prediction (same probability for both classes). A higher value corresponds to a higher influence on the final model answer

Further interpretation was performed by computing the Shapley values with the SHAP algorithm. Figure 7 represents the mean Shapley values computed on the test set for each variable in the model with corresponding confidence intervals (95%) computed by bootstrapping. This represents the mean effect in absolute value of each variable, that is, the expected change from the mean prediction (probability 0.5) for each variable. This assigns to the CRC total score variable the largest effect from all the selected variables, followed by PHP-9, Obesity and Change in employment status all with a similar importance.

**Fig. 8:**
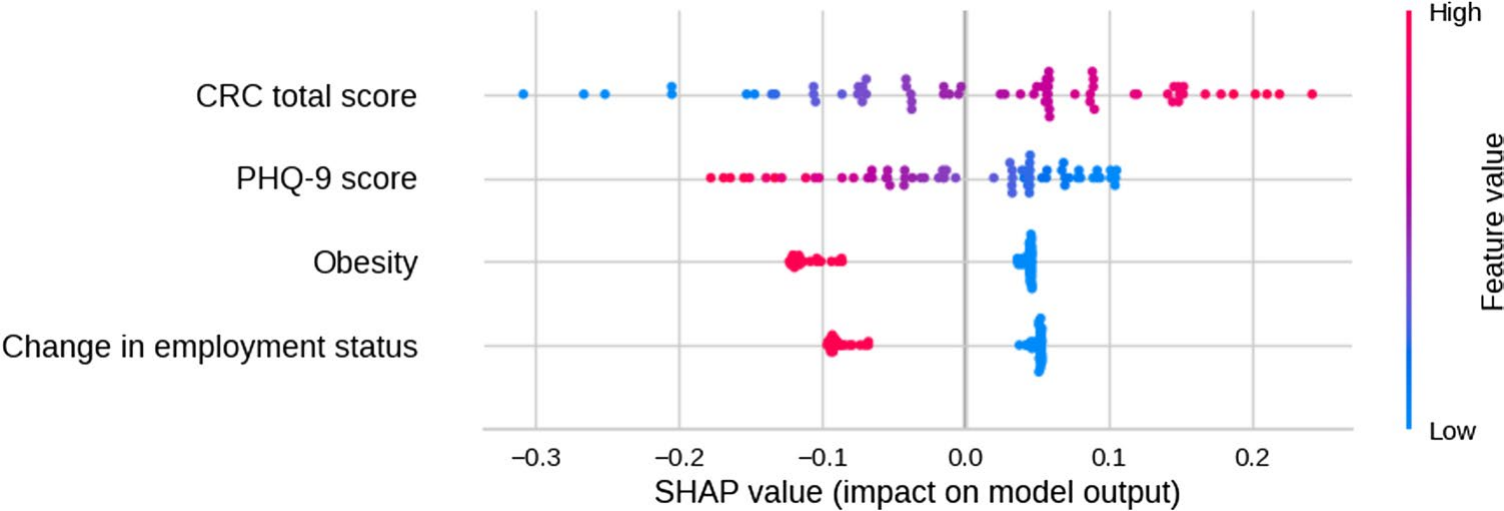
Distribution of the Shapley values of the test examples with respect to the magnitude of the data variables. CRC = Cognitive Reserve Questionnaire; PHQ-9 = Patient Health Questionnaire-9

A detailed analysis of the Shapley values for individual examples in the test data according to the magnitude of the variables is presented in Fig. 8. A higher CRC total score has a positive effect, moving examples toward the GP class, the effect seems distributed equally in both directions depending on the magnitude of its value, except for an extreme case. For the obesity variable, the impact of being obese has a larger effect on the BP class than not being obese. A similar trend can be observed for the change in employment status variable, although smaller. For the PHQ-9 total score variable, larger values lean the classification to the BP class, but the distribution of the effect is uneven, with higher values having more impact than lower values.

**Fig. 9:**
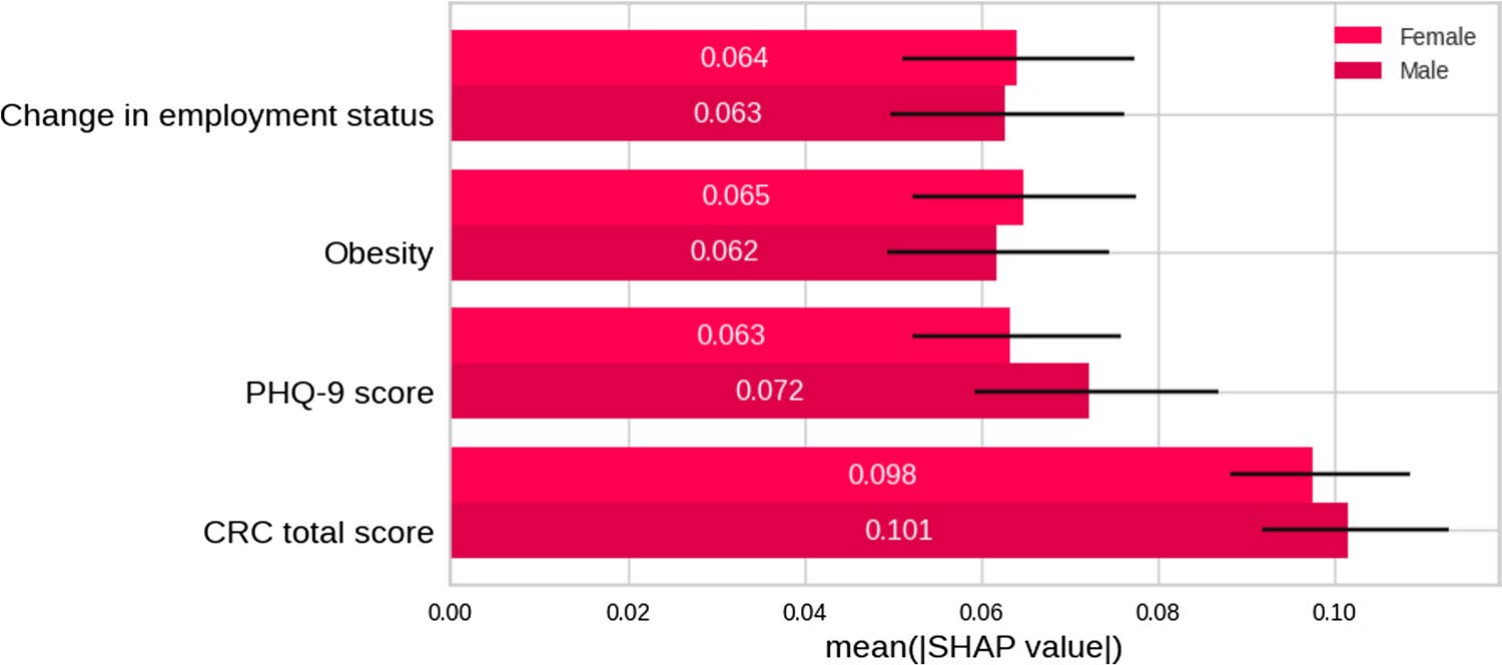
Shapley mean values ​​calculated for variables segregated by sex for each variable in the model. *CRC* Cognitive Reserve Questionnaire, *PHQ-9* Patient Health Questionnaire-9

**Fig. 10:**
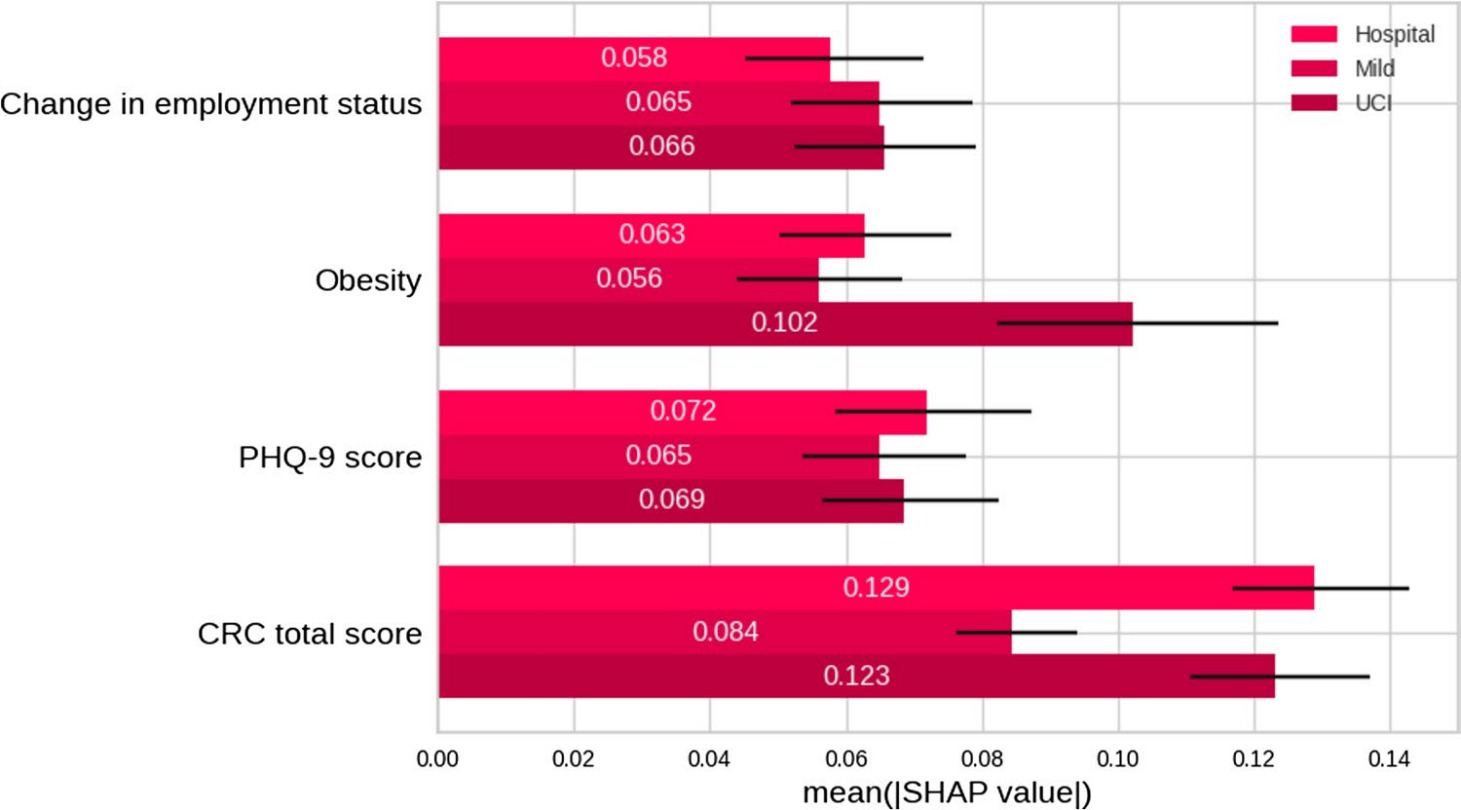
Shapley mean values ​​calculated for variables segregated by severity for each variable in the model. *CRC* Cognitive Reserve Questionnaire, *PHQ-9* Patient Health Questionnaire-9

A more detailed analysis of the relevance of the variables was performed by partitioning the dataset according to the sex and COVID severity variables, as represented in Figs. 9 and 10. There is no significant difference in the importance of the variables disaggregating the decisions by sex. For the severity disaggregation, there is a larger difference on the CRC total score and obesity variables. For the most severe cases (hospital and ICU), the CRC variable was more important. The obesity variable has greater importance for ICU cases.

## Discussion

The aim of this study was to identify the sociodemographic, clinical, and lifestyle characteristics that characterize a group of PCC participants with neuropsychological impairment. We focused on the performance of mental processing and executive dysfunction because alteration of these cognitive functions has been widely demonstrated in objective assessments [9, 50, 60–62], and in our previous research [17, 39].

Although cognitive complaints are one of the most common persistent symptoms of PCC [63], not all individuals confirm cognitive impairment. In many cases, other symptoms predominate. Matias-Guiu et al. [9] previously described the clustering of their sample based on the severity of cognitive impairment rather than cognitive phenotypes. The results of our cluster analysis revealed the presence of two distinct groups based on their different performance levels.

Based on the standardized results, it is evident that our BP group would fall within the cognitive impairment category. This classification is supported by the fact that all the tests, except Trail making Test (TMT) A, exhibit scores that are 1–1.5 standard deviations below (over in the case of TMT B) those of the GP group [9, 50]. Nevertheless, based on the PCA visualization, there is some degree of overlap between the groups. Consequently, while logistic regression can establish a boundary between the two classes, the separation may be rather ambiguous. Notwithstanding the oversimplification inherent in assuming linear separation between the two groups, the main trends in the relationship between variables should be valid. In addition, it was noted that all cognitive variables were of comparable importance in delineating these groups.

Our study revealed that individuals diagnosed with PCC who exhibit lower CR, more depressive symptoms, obesity, and changes in employment status were more at risk for experiencing BP in tasks related to mental speed processing and executive function.

CR was the variable with the most weight in predicting performance in mental processing speed and executive function. CR is a theoretical concept that refers to the adaptability and flexibility of cognitive processes and explains why cognitive abilities or functions are susceptible to deterioration with age, disease, or brain injury [64]. Variations in CR can be attributed to the characteristics of functional brain processes, which are shaped by the interaction between innate and environmental influences throughout life. As a theoretical construct, CR has often been assessed using lifestyle proxies, such as educational level, employment position, or participation in intellectually stimulating activities. Nevertheless, it is important to note that each proxy variable has a distinct impact on CR [64, 65], indicating that the approximations used do not fully encompass the entirety of the CR. One potential approach for addressing this issue involves the utilization of a comprehensive measure that considers several parameters, such as the CR questionnaire employed in our study [57].

CR is a protective factor in susceptibility to dementia [66, 67] and cognitive impairment resulting from acquired brain damage [68–70] or mental illnesses [71, 72]; a higher CR indicates a more robust capacity to cope with brain injury [73]. Our findings align with previous studies showing that CR is also the most significant protective factor for post-COVID cognitive impairment [24–27]. These studies have been performed with patients who required ICU care during COVID-19 infection. Navarra-Ventura’s study [24] found that a higher level of CR was positively correlated with improved working memory performance, and Fernandez-Gonzalo et al. [26] found that the cognitively normal cluster had a higher CR based on educational attainment and estimated IQ, 1 month after ICU discharge. In addition, Costas-Carrera et al. [27] reported that a higher CR was related to a reduced risk of developing verbal memory or executive function impairment 6 months after discharge, and CR emerged as a protective factor against objective cognitive deficit 12 months after ICU discharge according to the study by Godoy-González et al. [25]. Other authors have found similar results using the CR-proxy education attainment in participants during the short-time following ICU discharge [21]. Studies that included individuals of all severities found that a higher education level had a protective effect against cognitive decline 6 months after COVID-19 infection [20, 22] and in long-COVID individuals [9, 19]. The participants in our study underwent evaluation, on average, 11 months after contracting COVID-19. Those individuals within the BP cluster presented with a significantly lower CR than those in the GP cluster. It should be noted that their educational attainment and estimated IQ scores were also comparatively lower, so they were removed from the regression variables.

It is worth highlighting the difference in the contribution of CR segregated by severity of COVID-19 to the model. According to the data, CR was more important for those with PCC who were hospitalized (ICU and hospitalized) than those with mild COVID-19. This is unsurprising since brain insult in the acute phase is likely greater in more severe cases. Brain injury during COVID-19 has multiple causes, including direct virus damage to the brain vasculature (SARS-CoV2 can enter the brain through the blood–brain barrier damaged by endothelial injury or cytokines and chemokines generated during infection) [74, 75], vasodilation, edema, and even cerebral ischemia produced by severe hypoxia secondary to systemic inflammation [76] or coagulopathy [77], and severe hypoxia from respiratory failure or sepsis, which is frequently observed in critical patients [78].

The second factor that helped to shape our model was depressive symptoms. Post-COVID patients exhibit elevated levels of depression [79, 80]. Even though some studies have found only weak [61, 62] or nonexistent relationships [81], most of the previous research has demonstrated a clear connection between depressive symptomatology and cognitive impairment [39, 82–86]. Reported depressive symptoms are the variable that gave the best predictive performance in attention and information speed processing [83, 86] and executive function [83, 85].

The causal relationship between depression and cognitive impairment is currently unknown. People with severe depressive disorder frequently exhibit cognitive impairments [87], particularly in executive dysfunction [88]. As previous studies [83, 85, 86] and the present study have shown, depressive symptoms predict mental processing speed and executive function in people with PCC. However, several characteristic markers of people with PCC, such as increased cortisol levels or increased pro-inflammatory factors [5], are associated with depression [89]. Furthermore, the implication of chronic inflammation on the development of cognitive impairment seems increasingly plausible [90]. According to inflammatory models of neurodegeneration, systemic inflammation increases neuro-inflammation, which in turn affects cognition [91]. Consequently, the same inflammatory process that causes post-COVID syndrome is responsible for cognitive dysfunction and depressive symptoms.

Finally, it cannot be ruled out that post-COVID symptoms, such as cognitive challenges, may cause depression. More research is needed to draw accurate conclusions regarding the connection between depression and cognitive deficits.

In our model, at body mass index (BMI) cutoff of 30 kg/m^2^, that is, being obese or not, was also a significant factor in classifying patients with PCC according to their mental processing speed and executive function performance. There is a significant connection between obesity and the increased severity and mortality rate among COVID-19 patients [92]. Furthermore, neuropsychological studies have linked obesity with cognitive impairments; obesity increases the likelihood of developing mild cognitive impairment [93]. Executive function has been shown to be reduced in obese subjects [94], and greater visceral adiposity has been related to slower mental processing speed [95]. Neuroimaging studies reported altered connections across brain regions, structural abnormalities, and task-related prefrontal cortex dysfunction [96]. Hypoactivation of the prefrontal cortex has also been observed during executive function performance [97]. In addition, obese participants exhibited altered functional connectivity strength in the salience network (putamen nucleus), which is associated with a slower mental processing speed [98].

It is currently unclear how obesity affects cognition; however, it is thought to be through inflammatory mechanisms. Obesity is characterized by chronic systemic inflammation, which involves the aberrant production of cytokines, heightened levels of acute-phase reactants, and activation of a network of inflammatory signaling pathways [99]. Research suggests that low-grade systemic inflammation can mediate BMI and executive function deficits [100–102].

Obesity increases the likelihood of developing PCC [6]. Recent evidence indicates that adipose tissue appears to be one of the primary sources of increased levels of chemokines in PCC individuals [102] and that chronic inflammation may be an etiopathogenic factor of PCC (5). Our results also show that obesity is a risk factor for post-COVID cognitive impairment. That risk is probably increased by the additive effect of chronic inflammation to an inflammatory disease, such as COVID-19 [103]. As expected, the contribution of obesity to the severity segregation pattern of COVID-19 was more important in ICU cases, where the inflammatory processes are more severe.

Finally, change in employment status was also a significant predictor of poorer cognitive performance. Employment status is part of the social determinants of health, which are factors related to the conditions around an individual’s birth and living situations that influence their overall health [104]. An individual’s socioeconomic status is widely recognized as a significant determinant of their overall health. Individuals with lower socioeconomic status are more susceptible to experiencing premature mortality, developing disability, and dementia [105, 106]. It is also associated with good brain health and better aging. In a population sample of men without dementia, those with socioeconomic advantages demonstrated superior psychomotor function, visual learning, and general cognition [107].

Unemployment has been reported to be associated with COVID-19 mortality in Chilean [108] and North American [109] cohorts. Lower socioeconomic status is associated with a higher risk of developing persistent neurological symptoms [8]. Our results show that a change in employment status is important in classifying people with PCC based on their performance on tests assessing the processing speed and executive function. Furthermore, this change in the employment situation affected all labor groups, not just those with lower qualifications. People in intellectually demanding occupations experienced difficulties in continuing a satisfactory performance, became less productive, and felt obliged to resign or accept lower-skilled positions. People who are unable to return to work full-time usually undergo disease exacerbations, which necessitate an extended period of medical leave. These individuals’ incapability to restore themselves to their prior level of activity carries significant economic and social consequences.

In contrast to previous research [9, 22, 110, 111], our model revealed no effect of age or gender. In fact, the evidence for the direction of the relationship of age and gender with respect to cognitive impairment is inconclusive. Some authors reported that the risk of cognitive impairment increased with age [22, 111], whereas others found that the risk was greatest for the youngest individuals [9, 112]. Regarding gender, it has been determined that both males [22] and females [19] are at risk for cognitive impairment. In our participants, the duration between the onset of the disease and the evaluation was highly variable. However, in this case, the two clusters for this variable were comparable, meaning it had no explanatory value. Conversely, the cross-sectional design limits the optimal implementation of this analysis. A further investigation should be conducted into the influence of time on the recovery process from cognitive impairment following COVID-19.

Our model explains a substantial portion of the variance in cognitive performance between groups; however, it is not ideal. Structural and functional brain abnormalities in people with post-COVID cognitive complaints, likely of neuroinflammatory origin, have been linked to alterations in attention, processing speed, and working memory after 1 year [113]. Conversely, chronic inflammation, persistent SARS-CoV-2 viral antigens, and reactivation of inactive herpesviruses have been proposed as factors that contribute to the persistence of symptoms [5]. A further investigation should be conducted incorporating plasma markers of inflammation and structural and functional neuroimaging data, to acquire a more precise profile of the factors predicting cognitive impairment following COVID-19.

Our study’s limitations and strengths must be considered when forming conclusions. First, the cross-sectional nature of our study design prevented us from determining causal relationships with precision. Our partition cannot ensure that the low-performance cluster is entirely made up of people with psychometric impaired cognition, because the clusters overlap. We lost several cases that had to be excluded from the analyses due to missing values that could not be imputed. Finally, although this study was conducted across multiple centers, all of them maintained a consistent cultural environment. Replication in other samples should be performed to confirm the generalizability of these findings.

However, we recruited a large sample size, including patients from several health centers, representing the full spectrum of severity of COVID-19. In addition, the selection of the sample was performed by ruling out comorbidities that could cause cognitive impairment. Participants underwent a comprehensive assessment with multiple levels, including clinical, neuropsychological, emotional, and lifestyle dimensions.

To conclude, despite methodological limitations, we found that PCC individuals with a lower CR, greater depressive symptoms, obesity, and a change in employment status were at greater risk for poor performance on tasks requiring mental processing speed and executive function. This information allows for the identification of at-risk individuals and the development and implementation of early intervention programs. Some of these factors are modifiable. Promoting a healthy lifestyle, avoiding a sedentary lifestyle, and increasing physical activity, as well as adequate nutrition, engaging in novel and stimulating activities, and cognitive remediation are ways to increase CR, emphasizing the neuroplasticity-influencing factors in participants with PCC.

## Supplementary Information

Below is the link to the electronic supplementary material.

Supplementary file1 (DOCX 23 KB)

Supplementary file2 (DOCX 16 KB)

## Data Availability

The data that support the findings of this study are available from the corresponding author upon reasonable request.
