# Supplementary material for: Cognitive reserve, depressive symptoms, obesity, and change in employment status predict mental processing speed and executive function after COVID-19
Source: Eur Arch Psychiatry Clin Neurosci. 2024 Jan 29;275(4):973–89. doi: 10.1007/s00406-023-01748-x (PMC12148995; doi:10.1007/s00406-023-01748-x)
Supplement: Supplementary file 1 — Supplementary file1 (DOCX 23 KB) [file 406_2023_1748_MOESM1_ESM.docx]

**Supplementary Table 1. Post-COVID participant characteristics according to hospital of recruitment.**

|  | **CST** | **HUGTP** | **CSI-HUMB** | **HUSM-HUAV** | **CSG** | **HNSM** | **FSHSU** | **CHV** | **HCB** | **HTVC** | **HUB** | **HUMT** | **BSA** | **IAS** | **FSE** | **FHP** | **HCCR-SJSA** | **HSR** |
| --- | --- | --- | --- | --- | --- | --- | --- | --- | --- | --- | --- | --- | --- | --- | --- | --- | --- | --- |
| **N (%)** | | | | | | | | | | | | | | | | | | |
| **Total** | 122 (28.6%) | 7  (1.6%) | 32  (7.5%) | 79 (18.5%) | 15  (3.5%) | 10  (2.3%) | 10  (2.3%) | 27  (6.3%) | 6  (1.4%) | 18  (4.2%) | 11  (2.6%) | 17  (4%) | 17  (4%) | 25  (5.9%) | 12  (2.8%) | 6  (1.4%) | 6  (1.4%) | 6  (1.4%) |
| **Severity** |  |  |  |  |  |  |  |  |  |  |  |  |  |  |  |  |  |  |
| *ICU* | 39  (32%) | 0 | 8  (25%) | 23 (29%) | 1  (6.7%) | 2  (20%) | 0 | 4 (14.8%) | 0 | 10 (55.6%) | 0 | 3  (17.6%) | 0 | 2  (8%) | 1  (8.3%) | 0 | 0 | 3  (50%) |
| *Hospital* | 16 (13.1%) | 0 | 8  (25%) | 7  (8.9%) | 7 (46.7%) | 2  (20%) | 4  (40%) | 14 (51.9%) | 0 | 1  (5.6%) | 5 (45.5%) | 5 (29.4%) | 3 (17.6%) | 7  (28%) | 4 (33.3%) | 0 | 2 (33.3%) | 1 (16.7%) |
| *Mild* | 67 (54.9%) | 7 (100%) | 16 (50%) | 49 (62%) | 7 (46.7%) | 6  (60%) | 6  (60%) | 9 (33.3%) | 6 (100%) | 7 38.9(%) | 6 (54.5%) | 9 (52.9%) | 14 (82.4%) | 16  (64%) | 7 (58.3%) | 6  (100%) | 4 (66.7%) | 2 (33.3%) |
| **Sex (female)** | 82 (67.2%) | 6 (85.7%) | 16 (50%) | 52 (65.8%) | 14 (93.3%) | 7  (70%) | 7  (70%) | 16 (59.3%) | 6 (100%) | 13 (72.2%) | 4 (36.4%) | 10 (58.8%) | 11 (64.7%) | 17 (68%) | 9  (70%) | 5 (83.3%) | 4 (66.7%) | 6 (100%) |
| **Mean (SD)** | | | | | | | | | | | | | | | | | | |
| **Age (years)** | 51  (7.2) | 48.3 (8.8) | 52  (8.4) | 51.7 (9.4) | 45.4 (9.4) | 50.7 (10) | 52.6 (6.9) | 52.1 (11.1) | 51.1 (7.9) | 49.8 (13.1) | 49.7 (9.8) | 46.7 (11.3) | 47.9 (7.8) | 51.6  (8) | 47 (11.2) | 48.7 (6.3) | 42.3 (11.1) | 40.8 (19.6) |
| **Education (years)** | 13.8  (3.4) | 15.1 (3.3) | 14.6 (4.3) | 13.82 (2.7) | 13.9 (3.9) | 13.9 (3.6) | 12.1 (2.3) | 13.9 (3.5) | 15.6 (3.5) | 12.8 (3.4) | 13.7 (3.2) | 14.8 (3.5) | 14  (3.2) | 12.7 (3.7) | 14.8 (3.6) | 15  (1.3) | 15.7 (2.2) | 17.2 (6.6) |
| **Time since**  **onset to assessment (days)** | 436  (284) | 428 (333) | 396 (259) | 365 (224) | 290 (132) | 285 (174%) | 373 (115) | 266  (81) | 403 (278) | 271 (105) | 290 (174) | 345 (174) | 308 (166) | 311 (144) | 252  (73) | 367  (155) | 215 (123) | 408 (319) |

CST= Consorci Sanitari de Terrassa (Terrassa, Barcelona, Spain); HUGTP= Hospital Universitari Germans Trias i Pujol (Badalona, Barcelona, Spain); CSI-HUMB= Hospital Sant Joan Despí Moisès Broggi- Consorci Sanitari Integral (Sant Joan Despí, Barcelona, Spain), HUSM-HUAV= Hospital Universitari de Santa Maria (Lleida, Spain) and Hospital Universitari Arnau de Vilanova (Lleida, Spain); CSG= Consorci Sanitari Alt Penedès-Garraf (Vilafranca de Penedés, Barcelona, Spain); HNSM= Hospital Nostra Senyora de Meritxell (Andorra); FSHSU= Fundació Sant Hospital (La Seu d’Urgell, Lleida, Spain); CHV= Consorci Hospitalari de Vic (Vic, Barcelona, Spain); HCB= Hospital Clinic de Barcelona (Barcelona, Spain); HTVC= Hospital Verge de la Cinta, (Tortosa, Tarragona, Spain); HUB= Hospital Universitari de Bellvitge (Barcelona, Spain); HUMT= Hospital Universitari Mútua de Terrassa (Terrassa, Barcelona, Spain); BSA= Hospital Municipal Badalona (Badalona, Barcelona, Spain); IAS= Institut d'Assistència Sanitària (Girona, Spain); FSE= Hospital de Figueres (Figueres, Girona, Spain); FHP= Hospital de Puigcerdà (Puigcerdà, Girona, Spain); HGCR-SJSA= Hospital General de la Cruz Roja San José y Santa Adela (Madrid, Spain); HSR= Hospitales San Roque (Gran Canaria, Islas Canarias, Spain).
