# Supplementary material for: Cognitive reserve, depressive symptoms, obesity, and change in employment status predict mental processing speed and executive function after COVID-19
Source: Eur Arch Psychiatry Clin Neurosci. 2024 Jan 29;275(4):973–89. doi: 10.1007/s00406-023-01748-x (PMC12148995; doi:10.1007/s00406-023-01748-x)
Supplement: Supplementary file 2 — Supplementary file2 (DOCX 16 KB) [file 406_2023_1748_MOESM2_ESM.docx]

Supplementary Table 2. Distribution of post-COVID-19 participants by occupational group prior to COVID-19 disease

|  | **Occupational group** |
| --- | --- |
| Directors and managers | 38 (9.1%) |
| Health and teaching scientific and intellectual technicians and professionals | 75 (17.9%) |
| Other scientific and intellectual technicians and professionals | 21 (5.0%) |
| Technicians and support professionals (science and engineering technicians) | 22 (5.3%) |
| Accounting, administrative and other office employees | 95 (22.7%) |
| Restaurant industry, personal protection and sales services | 1 (0.2%) |
| Health and personal care | 51 (12.2) |
| Protection and security services | 11 (2.6%) |
| Qualified workers in agriculture, forestry and fishing industry | 5 (1.2%) |
| Craftsmen and skilled workers in manufacturing | 10 (2.4%) |
| Skilled workers in manufacturing industries | 8 (1.9%) |
| Plant and machine operators and assemblers | 7 (1.8%) |
| Drivers and mobile-plant operators | 11 (2.6%) |
| Elementary occupations: unskilled workers in services | 41 (9.8%) |
| Laborers in agriculture, fishing, construction, manufacturing industries and transportation | 9 (2.1%) |

Supplementary Table 3. Frequency of change in employment status by occupational group after COVID-19 disease

|  | **Employment status** | |
| --- | --- | --- |
|  | **No change**  N= 224 | **Change**  N= 181 |
| Directors and managers | 27 (71.1%) | 11 (28.9%) |
| Health and teaching scientific and intellectual technicians and professionals | 43 (57.3%) | 32 (42.7%) |
| Other scientific and intellectual technicians and professionals | 10 (47.6%) | 11 (52.4%) |
| Technicians and support professionals (science and engineering technicians) | 12 (54.5%) | 10 (45.5%) |
| Accounting, administrative and other office employees | 47 (49.5%) | 48 (50.5%) |
| Restaurant industry, personal protection and sales services | 1 (100%) | 0 |
| Health and personal care | 32 (62.7%) | 19 (37.3%) |
| Protection and security services | 4 (36.4%) | 7 (63.6%) |
| Qualified workers in agriculture, forestry and fishing industry | 4 (80%) | 1 (20%) |
| Craftsmen and skilled workers in manufacturing | 6 (60%) | 4 (40%) |
| Skilled workers in manufacturing industries | 5 (62.5%) | 3 (37.5%) |
| Plant and machine operators and assemblers | 7 (100%) | 0 |
| Drivers and mobile-plant operators | 2 (18%) | 9 (82%) |
| Elementary occupations: unskilled workers in services | 20 (49%) | 21(51%) |
| Laborers in agriculture, fishing, construction, manufacturing industries and transportation | 4 (44.4%) | 5 (56.6%) |
